# Responsiveness and Reliability of a Sipping Device to Measure Motivation in Normal-Weight Individuals and Bariatric Surgery Patients

**DOI:** 10.3390/nu16173001

**Published:** 2024-09-05

**Authors:** Jeon D. Hamm, Blandine Laferrère, Jeanine B. Albu, Subhash Kini, Xavier Pi-Sunyer, Harry R. Kissileff

**Affiliations:** 1 Howard University College of Medicine, Washington, DC 20059, USA; jeon.hamm@mountsinai.org; 2 Diabetes, Obesity, & Metabolism Institute, Icahn School of Medicine at Mount Sinai, New York, NY 10029, USA; 3 Division of Endocrinology, Columbia University Irving Medical Center, New York, NY 10032, USA; bbl14@cumc.columbia.edu (B.L.); fxp1@cumc.columbia.edu (X.P.-S.); 4 Division of Endocrinology, Department of Medicine, Mount Sinai Morningside Hospital, New York, NY 10029, USA; jeanine.albumd@mountsinai.org; 5 Institute of Bariatric and Minimally Invasive Surgery, Mount Sinai Morningside Hospital, New York, NY 10025, USA; subhash.kini@mountsinai.org

**Keywords:** Motivation, sweet-taste, progressive-ratio, bariatric surgery

## Abstract

There is an urgent need to measure the motivation to taste a sweet fluid in order to determine the influence of sweet tastes on the potential choices and consumption of beverages in patients with obesity. Current methods utilize either survey instruments or arbitrary operant tasks. The sipometer enables the participant to utilize an actual ingestive behavioral response to measure motivation during access to beverages on either ad libitum (AL) or progressive time ratio (PR) schedules. We determined the sipometer’s responsiveness and reliability as a test of change in motivation for sweet tastes after bariatric surgery. Participants (58 patients and 28 controls, BMI: 18.5–24.9 kg/m^2^) sham-consumed an aspartame-sweetened (S) and non-sweetened (N) beverage under AL and PR schedules at a pre-surgery/baseline and a 3-month and 24-month visit (patients only). Cumulative pressure (CumPres), a measure of effort, was the sum of the pressures exerted during sipping under each condition. Baseline CumPres for PRS was higher than ALS and ALN in patients (*p* < 0.03) and higher than PRN in controls (*p* = 0.009). At 3 months, CumPres did not differ amongst conditions in patients, but CumPres for PRS was higher than all other conditions in controls (*p* < 0.0005). There were no baseline group differences; however, patients’ CumPres for PRS was lower than controls’ at 3 months (*p* = 0.002). Patients’ CumPres for PRS decreased non-significantly between the baseline and 3 months but increased at 24 months compared to 3 months (*p* = 0.025) and was no different from baseline. Controls’ CumPres for PRS increased at 3 months (*p* = 0.0359), but CumPres for all conditions was correlated between visits (*p*’s < 0.038). The sipometer is a reliable and sensitive measure of motivation to consume sweet beverages and may reflect changes in post-operative energy intake.

## 1. Introduction

### 1.1. Background

#### 1.1.1. Sweet Taste—Nutritive Versus Nonnutritive Sweeteners for Motivational Assessment

An enduring problem in behavioral neuroscience is the source of the reinforcing stimulus in a reinforcer. Early theories of learning and motivation (e.g., Hull, Miller) postulated that reward only occurred when the drive was reduced, so that a sweet taste, for example, would not be motivating (i.e., generate an effort to obtain it) unless there was a drive to be reduced [1,2]. This theory’s limitation was shown when Sheffield, Roby, and Campbell [3] demonstrated that a reward could occur in the absence of need reduction since the reinforcer had virtually no nutritive value. This demonstration introduced the field to the use of sweet taste as a reward in its own right, reviewed and labeled with neurological underpinnings by Pfaffmann as the “pleasures of sensation” [4]. The incentive value of saccharin as a reinforcer was further shown in a dose-response study in which deprived and non-deprived rats’ bars were pressed at similar rates for different saccharin concentrations [5]. There are now a host of such products, all low-calorie sweeteners (LCS), and their sweet taste properties differ with the chemical structures of the specific sweeteners and with differing species. Rats, for example, have very little interest in aspartame [6], whereas for humans, aspartame is sweeter than saccharin [7] and lacks the bitter component at high intensity.

The current report highlights the use of aspartame as an LCS and motivational stimulus in humans without supplying any nutritional value at all by means of a sham-drinking paradigm. It is included in this special issue to illustrate the use of an LCS as a component of motivational assessment related to human health. Aspartame was used for the comparison of motivation to taste sweetness between healthy individuals and those with obesity who undergo bariatric surgery.

This paradigm was based on previous work with eating-disordered patients [8] in which a sham-drinking paradigm was used to prevent the influence of the post-ingestion effects on the response to obtain a sweet taste. In an earlier reported study in the same individuals used in the present report, several concentrations of both nutritional and LCSs were tested and included concentrations above and below the 10% sucrose concentration typically used in commercial beverages [9]. We found that a 10% sucrose-equivalent aspartame-sweetened, flavored beverage was wanted more than those of lower concentrations [9]. The desire to consume this beverage was sufficient to support the effort to obtain it [10] despite its low-calorie value, which warranted its use in the present study. Previous studies [11,12] on the motivational properties of aspartame in humans have relied on questionnaires [13], while the current study uses an effort-requiring task.

#### 1.1.2. Motivation to Work for Sweets

Motivation to work for and consume sweet items has long been studied [14]. The technique used in the current study [10,15] was based on an animal study by Sclafani and Ackroff that showed that a progressive ratio operant licking task measures the motivation for sweet drinks (sucrose) [16]. The increased motivation was obscured by post-ingestive effects on a fixed ratio schedule as licks increased and then decreased as the sucrose concentration increased; however, the relationship was linear on a progressive ratio schedule (PR) [16].

#### 1.1.3. Motivation to Work for and Consume Sweets in the Context of Bariatric Surgery in Animals and Humans

Bariatric surgery reduces food intake and promotes weight loss, but the role of changes in taste responsiveness as a motivational stimulus is controversial [17]. Some animal studies (e.g., Hajnal et al. [18]) reported a reduced preference for sweet tastes, while others found no evidence that bypass surgery in rats influenced orosensory properties of sucrose that motivate concentration-dependent licking in brief access tests [19]. Corresponding human studies of taste perception after Roux-en-Y gastric bypass (RYGB) and vertical sleeve gastrectomy (VSG) surgeries demonstrated a post-operative shift in sweetness palatability from pleasant to unpleasant under repeated tasting of sucrose [20,21]. Further, some studies of sensory thresholds in humans showed that recognition thresholds for sucrose, but not other tastants, were reduced after bypass surgery [22]. These results were confirmed by Bueter et al. [23]; however, in their study, suprathreshold hedonic ratings were unchanged up to a concentration of 0.4 M. This result accords with the findings of Bray et al., who demonstrated that decreases in pleasure after surgery were only found at 40% (c. 1.2 M sucrose) [24].

Miras et al. demonstrated that after bariatric surgery in humans, patients were less motivated to work for a sweet-tasting candy [25]. The median breakpoint for candies, but not vegetables, was reduced by 50% in the obese group after gastric bypass, and patients with the largest reduction in the breakpoint had the largest decrease in body mass index [25]. Another potential indicator of motivation is the size of the meal. Gero and Bueter found that meals measured with a drinkometer were smaller after bypass surgery in humans [26]. Geary et al. found that more than six months after Roux-en-Y gastric bypass surgery, artificially sweetened diet meal sizes were larger in Roux-en-Y gastric bypass rats than sham rats, whereas Ensure^®^ meals were smaller [27]. These studies point to the motivation to consume sweets as an important research topic in regard to differences in post-operative outcomes in bariatric surgery patients.

#### 1.1.4. Novelty of the Sipometer

Classically, motivation has been studied by means of the progressive ratio (PR) task [28]. The progressive ratio task extended to the current study has been used in several studies, in various clinical populations, with reinforcers such as M&Ms [29] or as a strawberry yogurt shake [30], with a reward gained during [31] or at the end [30,32] of the session. Our approach was to utilize a sipping device called the ‘sipometer’ [10] to allow participants to obtain the reward as they earn it, with the reward being a sweet-tasting stimulus.

In order to study motivation to work for sweet drinks, specifically before and after bariatric surgery, we used the sipometer, which has been validated in ‘healthy’ individuals by Hogenkamp et al. [10] but has neither been validated in people who undergo bariatric surgery nor in repeated trials under the same conditions in healthy individuals. Prior studies of motivation utilized break points in the progressive ratio schedule as a measure of reinforcement. However, that measure is confounded with the duration and persistence and not necessarily with the intensity of the motivation to perform. In the current study, the measure of motivation was actual physical work, the amount of cumulative pressure exerted on a straw to obtain the sweet drink reinforcer.

### 1.2. Aims, Hypothesis, and Expected Outcomes

#### 1.2.1. Aims

The aims of this report are to (1) demonstrate motivational (i.e., effort-requiring) versus simple consummatory responsiveness of the sipometer in bariatric surgery patients and (2) demonstrate the reliability of the sipometer in non-surgical, normal-BMI controls.

#### 1.2.2. Hypothesis

The overarching hypothesis is that responding is a function of the schedule of reinforcement and taste of the reinforcer and that a sweet taste is more rewarding than a non-sweet taste. As was the case in the earlier study [10], we used two schedules of reinforcement (progressive ratio = PR and ad libitum = AL—see Section 2 for details) and two fluid reinforcements, a sweet and non-sweet beverage.

#### 1.2.3. Expected Outcomes

##### Aim 1

To address the first aim of responsiveness, we expected the six outcomes expressed below.

Expected outcomes one and two—condition differences. Hogenkamp et al. found that in healthy-weight individuals, the sipping time was longer for sweet than non-sweet beverages on a PR schedule but not ad libitum (AL) schedule [10]. Therefore, we expected that (E1) responding to obtain a sweet taste will be greater when effort is required (i.e., on a PR schedule) than when the fluid is AL in study participants at the baseline. Additionally, (E2) we expected that there would be no difference in responding between schedules (AL vs. PR) to obtain the non-sweet reinforcer in participants because the non-sweet lacks the reward value necessary to elicit motivation to obtain it [10].

Expected outcomes three, four, and five—visit differences. It has been reported that motivation to consume sweet tastes is attenuated post-operatively in bariatric patients [25,26]. Therefore, we expected that (E3) in patients after surgery, responses would decrease on PR for sweet but not for non-sweet tastes, whereas in controls over time, there would be no change in PR response for either sweet or non-sweet tastes. Under non-effort requiring tasks (AL) (E4), we expected that regardless of taste, responses would not change in patients after bariatric surgery or in controls over time. Moreover, because we did not expect patients’ responses for non-sweet, regardless of schedule, or sweet tastes without requiring work (AL) to change across visits, (E5) we expected that patients’ responses would correlate across visits for each condition more strongly for non-sweet tastes and non-work conditions than for the sweet, motivational conditions, i.e., PRS response, whose slope across visits would be reduced compared to the other three conditions.

Expected outcomes six and seven—group differences. While consumption of sweet, highly palatable items often leads to overconsumption and, over time, obesity [33], the literature does not consistently show that people with obesity (BMI > 30 kg/m^2^) have higher preferences for sweet tastes than people of ‘normal weight’ [34]. Therefore, (E6) we did not expect a difference between patients’ and controls’ responses to sweet taste during effort-requiring tasks (i.e., PR) at the baseline. However, (E7) we did expect that patients’ PR responses for sweet tastes would be lower than the controls at follow-up because, as mentioned above, patients are less motivated by sweet tastes after surgery than before surgery [25,26].

##### Aim 2

Expected outcomes eight and nine—control’s stability of response. To address the second aim on reliability in controls, we expected that (E8) responses on both schedules for sweet and non-sweet liquids would not change over time in the controls and (E9) these responses would correlate with each other.

## 2. Materials and Methods

### 2.1. Participants

Participants were recruited between May 2016 and April 2018. Individuals scheduled to undergo Roux-en-Y gastric bypass or vertical sleeve gastrectomy surgery (patients, *n* = 69) were recruited and consented 1–2 weeks before their scheduled surgery at Mt. Sinai–Morningside Hospital (New York, NY, USA). Individuals with a normal BMI (18.5–24.9 kg/m^2^) and of similar race, ethnicity, and age to the patients (controls, *n* = 30) responded to classified ads online (Columbia|RecruitMe database and Craigslist.org, San Francisco, CA, USA) or flyers posted around upper Manhattan and the Bronx, NY, and were consented at Columbia University Irving Medical Center (New York, NY, USA). Participants were selected for their rating of liking of a chocolate flavored Ensure^®^ (Abbott Laboraries, Green Oaks, IL, USA) during a ‘taste and spit test’ [9].

### 2.2. Study Design

This study was a smaller study within a larger study about pre-surgical psycho-behavioral predictors of post-surgical weight loss (NIH/NIDDK R01 DK108643).

**Figure 1:**
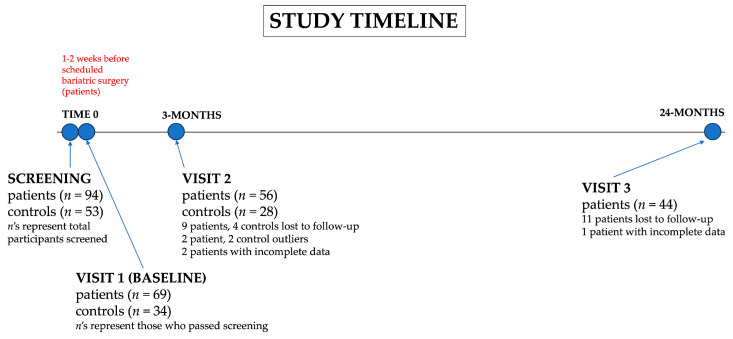
Study design and timeline. Below each visit, there is a brief explanation of the changes in sample size as the study progressed.

All study participants were scheduled for their first, or baseline, visit after being recruited for the study (1–2 weeks before scheduled Roux-en-Y gastric bypass or sleeve gastrectomy surgery in patients) and a second visit three months later. Patients returned for a third visit, which was 24 months after surgery. The sipometer task (described in Section 2.3) was completed at each visit (three visits in patients and two visits in controls). See Figure 1 for the study design and sample sizes at each visit. Note: This study was a smaller study than a larger study about pre-surgical psycho-behavioral predictors of post-surgical weight loss.

### 2.3. Instrumentation and Methods

**Figure 2:**
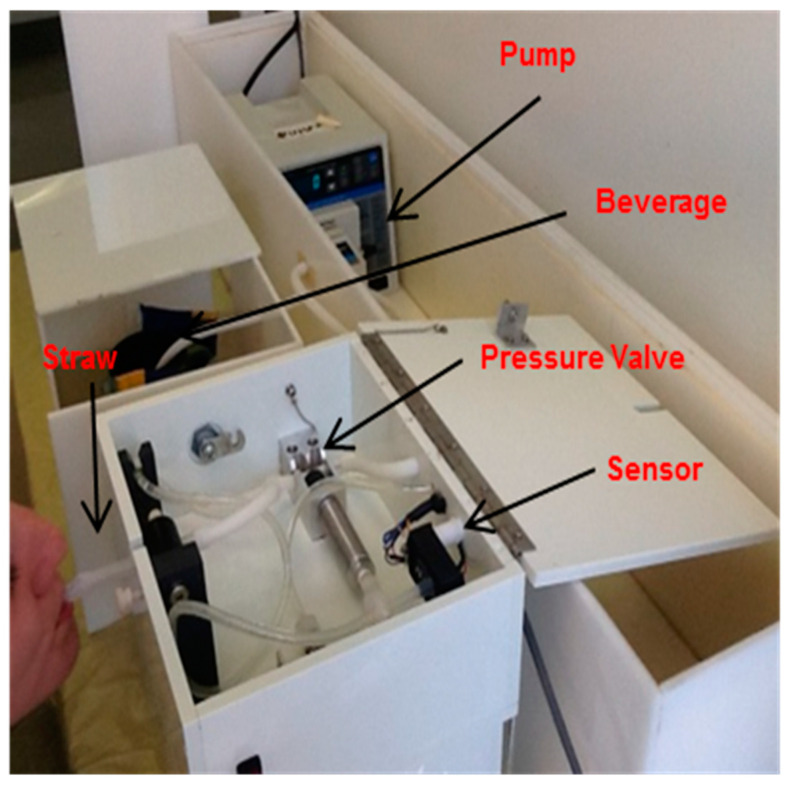
Sipometer schematic. The participant sips on the straw, and the pressure exerted is registered by the pressure sensor, which transmits the pressure reading to a computer in an adjacent room. When the criterion duration of sipping above minimal pressure is reached the pump delivers the liquid from the reservoir, through the tubing and straw until 7 mL has been disbursed within 2 s. The pressure value closure ensures that there is a minimal distance for measurement of pressure within the tubing system and no pressure drop between deliveries.

The sipometer is a sipping device that was developed to permit access to a liquid food or beverage in a laboratory setting [10,15]. The sipometer was used in the current study to measure motivation. By motivation, we mean the strength of the response measured by the pressure exerted needed to obtain access to a sweet or non-sweet-tasting liquid. Figure 2 shows a schematic of the key components of this instrument. The participant sipped on the straw, and the pressure exerted was detected by the pressure sensor and transmitted to a computer in an adjacent room. When the criterion response was completed, the pump was activated to deliver the beverage from the reservoir through the tubing and straw until 7 mL had been disbursed in 2 s. To enable the measurement of pressure when the fluid was not being delivered, a silent pressure-operated valve was placed in the system between the responder and the fluid source. After receiving the aliquot of beverage, the participants spat the contents of their mouths into a cup. The participant went back to the straw to work on more of the beverage. Instructions (scripts are in Supplementary Materials) for each trial were provided through speakers in the study room. Between trials that included different beverages, the research assistant pumped water through the system to clear the first beverage and pumped a sufficient amount of the second beverage so the beverage was positioned for delivery at the next trial. Between all trials, the participant also rinsed their mouth with distilled water.

The two beverages used in this experiment were cherry-flavored Kool-Aid (Kraft Foods, Inc., Northfield, MN, USA) that was either aspartame-sweetened (10% sucrose equivalent in sweet intensity, S) or non-sweetened (N) and served at 50 F in insulated containers. The 10% sucrose equivalent sweetened Kool-Aid solution was prepared by dissolving 0.375 g of aspartame in a 500 mL volumetric flask with distilled water plus 0.951 g of cherry-flavored unsweetened Kool-Aid [10]. The non-sweetened version was made by dissolving the same amount of cherry-flavored Kool-Aid in distilled water without the aspartame [10].

Cherry Kool-Aid flavor has long been used in many rodent studies of flavor preference conditioning since at least 1975. In rats, cherry was found to be iso-preferred to grape Kool-Aid, which is another commonly used flavor [35]. Further, cherry Kool-Aid has also been widely used in human taste and feeding studies [8,36,37,38,39,40,41,42,43,44]. One human study used both cherry and grape and reported that the ratings for the two flavors did not significantly differ [42].

The two sipping schedules were ad libitum (AL) and progressive time ratio (PR). Under the AL schedule, the participant was able to sip and spit each beverage freely for two minutes. No significant work was required to access the beverage aside from an initial pressure reading of 0.5 psi. Under the PR schedule, the participant was able to sip and spit each beverage for as long as they wanted; however, each additional sip required an additional 3 s of pressure exertion (minimum of 0.5 psi pressure exertion during each attempt) on the straw in order to receive the aliquot. The trials were counterbalanced, so within each beverage presentation, half the participants in each group received the S beverage first, with PR and AL counterbalanced, followed by the N beverage, with PR and AL counterbalanced. The other half of the participants in each group received the N beverage, followed by the S beverage, which was counterbalanced with PR and AL.

### 2.4. Data Collection

**Figure 3:**
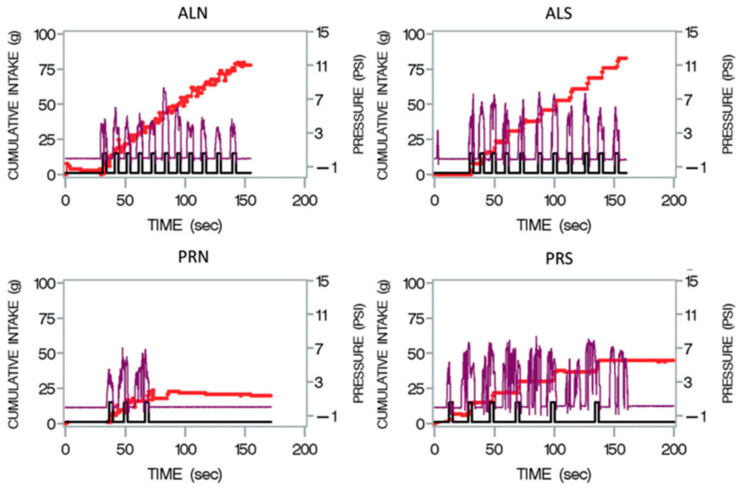
Four ‘sipographs’ [45] for one participant. The sipograph allows us to visualize the intake (red line), reinforcement (black line), and pressure exerted for each reinforcement (purple line). ALN, ad libitum schedule with non-sweet beverage. ALS, ad libitum schedule with sweet beverage. PRN, progressive ratio schedule with non-sweet beverage. PRS, progressive ratio schedule with sweet beverage.

**Figure 4:**
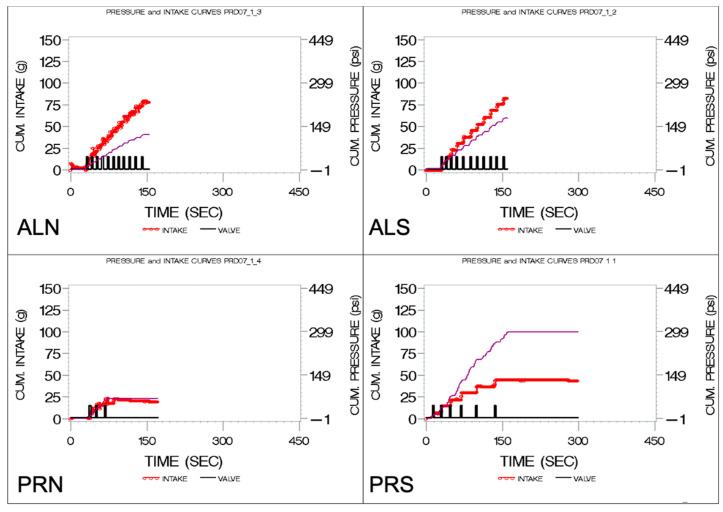
Graphs showing pressure (CUM PRESSURE) and intake curves (CUM. INTAKE) under 4 conditions. The graphs show the cumulative intake (red line), reinforcement (black line), and cumulative pressure exerted throughout the trial (purple line). ALN, ad libitum schedule with non-sweet beverage. ALS, ad libitum schedule with sweet beverage. PRN, progressive ratio schedule with non-sweet beverage. PRS, progressive ratio schedule with sweet beverage.

Output from the sipometer (pressure, intake, and reinforcement) was recorded in real time by a MATLAB R2020b program. Our measure of motivation was a derived variable called cumulative pressure (CumPres). To understand the CumPres, we will first graphically show the components of each trial with the sipometer. In Figure 3, this ‘sipograph’ [45] allows us to visualize the intake (red line), reinforcement (black line), and pressure exerted for each reinforcement (purple line). In order to more clearly see the pressure effects, the individual upward excursions were summed in a computer program written in SAS 9.4 by Kissileff (co-author) to produce the total, or CumPres, which is shown by the purple lines in Figure 4.

### 2.5. Data Analyses

All statistical analyses were performed in SAS 9.4 (SAS Institute, Inc., Cary, NC, USA).

Descriptive statistics were calculated for means and standard errors of the means across conditions (ALN, ALS, PRN, PRS), groups (patient or control), and visits (1–3). Independent *t*-tests were conducted on the age, BMI, and baseline weight of patients and controls. In order to ensure that differences among demographic variables were not primary influences on the outcomes, Kolmogorov–Smirnov 2-sample non-parametric tests were conducted to determine that cumulative pressures were not different across demographic variables. After confirmation (non-significant D statistics), the data were combined across sex, surgery type, group, ethnicity, and race and then used for multiple planned comparisons.

Two ANOVAs were conducted to detect differences, by planned comparisons, in cumulative pressures across conditions, groups, and visits, which would illustrate the responsiveness (Aim 1, E1–E7) and reliability (Aim 2, E8) of the instrument. In the first ANOVA, proc mixed was used with repeated measures on visits and sipping conditions, with patients and controls as between-group variables. Cumulative pressure was the dependent variable. The independent variables were (1) conditions at four levels (ALN, ALS, PRN, PRS), (2) groups (patients, controls), and (3) visits (baseline/pre-surgery = 1, 3 months later = 2). For analyses concerning patients and controls at visits 1 and 2, all participants who had complete data were included. Two patient and two control participants were Cook’s-D outliers and were removed from analyses. In the second ANOVA, similar to the first ANOVA, proc mixed was used with repeated measures on visits and conditions; however, this model only included patient data across the three visits. Proc mixed was used, and, once again, the dependent variable was cumulative pressure and the independent variables were visits and conditions. This analysis included a smaller *n* due to the participant attrition, or incomplete data, by the 24-month follow-up. All analyses used Tukey adjustment.

Linear regressions of cumulative pressures for visit 2 from visit 1, for each condition, were conducted to illustrate test–retest reliability (Aim 2, E9) by means of R^2^ in controls. Additionally, linear regressions of cumulative pressures at (1) visit 2 from visit 1, (2) visit 3 from visit 2, and (3) visit 3 from visit 1 were conducted in patients to further illustrate changes in response over time and how they relate to one another with significance set α = 0.05.

## 3. Results

### 3.1. Participant Characteristics

The Baseline weight and BMI were higher in patients than in controls (*p* < 0.0001) (Table 1), but age was not different (*p* = 0.72). Participants were primarily female (89% of patients and 79% of controls), Black (55% of patients and 61% of controls), and of Hispanic ethnicity (64% of patients and 68% of controls). Most patients underwent the vertical sleeve gastrectomy (62% VSG, 38% Roux-en-Y gastric bypass). There were no significant differences in participant response based on sex, race, ethnicity, and surgery type (D-statistics < 0.43, *p*’s > 0.05).

### 3.2. ANOVA Model 1—Differences across Groups, Visits, and Conditions (Schedule × Beverage)

#### 3.2.1. Overall Model

The overall model for the first ANOVA conducted, which included responses as the dependent variable and the group, visit, and condition (beverage × schedule) as the independent variables, was significant (F-statistic = 3.97, R^2^ = 0.40, Coefficient of Variation = 78.6, *p* < 0.0001). Group, visit, and condition effects were not significant (*p* > 0.05, Table 2). Group × visit (*p* < 0.0001) and group × condition (*p* = 0.0062) interactions were significant (Table 2), while visit × condition and group × visit × condition interactions were not (*p*’s > 0.05, Table 2). Differences and specific interactions presented in subsequent subsections of Section 3.2 are derived from this model.

#### 3.2.2. Differences across Conditions at Baseline (E1 and E2)

**Figure 5:**
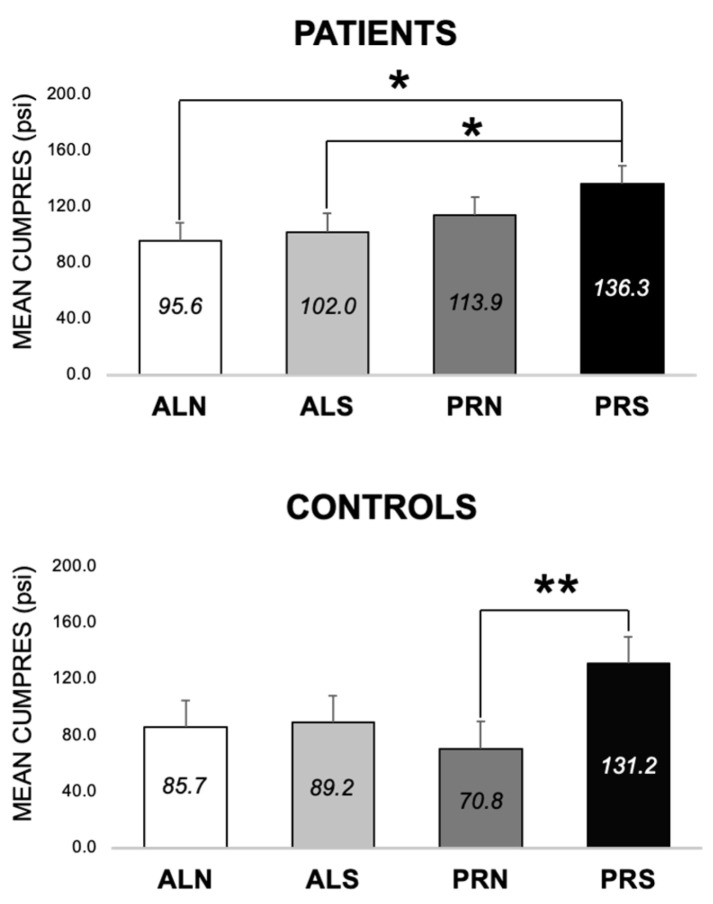
Mean cumulative pressure (MEAN CUMPRES) across four conditions (schedule × beverage) at baseline, for patients (*n* = 56) and controls (*n* = 28). Means are shown in each bar with the error bars of the standard error. * Indicates *p* < 0.03. ** indicates *p* < 0.01. ALN, ad libitum schedule with non-sweet beverage. ALS, ad libitum schedule with sweet beverage. PRN, progressive ratio schedule with non-sweet beverage. PRS, progressive ratio schedule with sweet beverage.

In patients, CumPres for PRS was higher than ALN by 40.6 ± 15.8 psi (*p* = 0.01) and ALS by 34.2 ± 15.8 psi (*p* = 0.03) but was not different from PRN (*p* > 0.05) (Figure 5). In controls, CumPres of PRS was higher than PRN by 60.4 ± 23.2 psi (*p* = 0.009) but was not different from ALN or ALS (*p* > 0.05) (Figure 5). Patients and controls’ CumPres across conditions followed a similar pattern of PRS, eliciting the most work from participants compared to other conditions.

#### 3.2.3. Differences across Conditions at 3-Month Follow-Up (E1 and E2)

**Figure 6:**
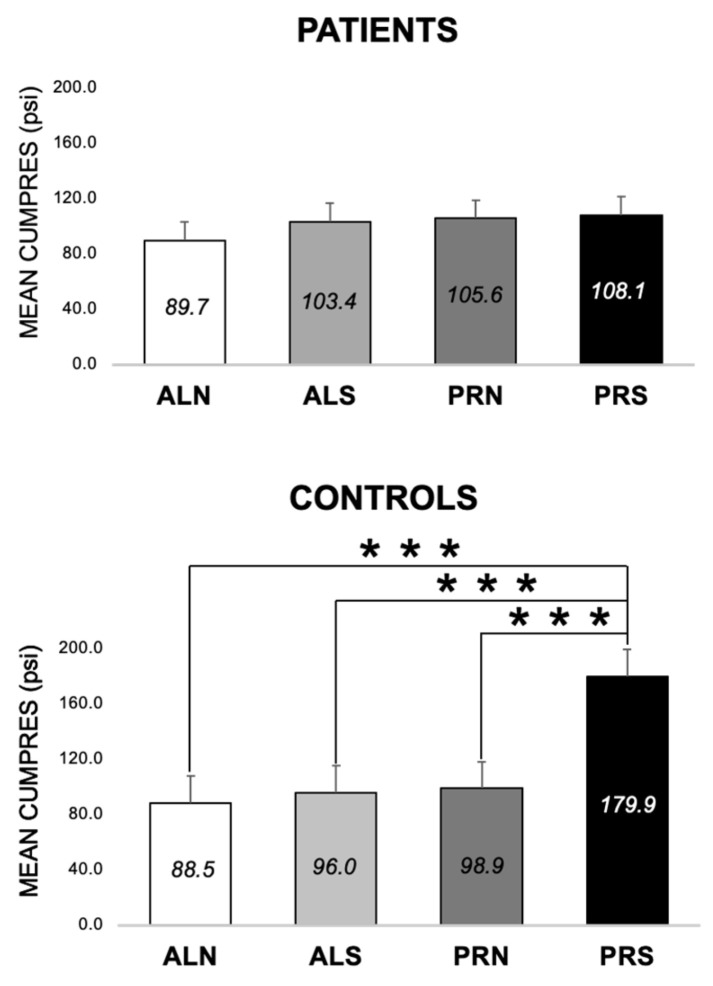
Mean cumulative pressure (MEAN CUMPRES) across four conditions (schedule × beverage) at the 3-month follow-up visit, for patients (*n* = 56) and controls (*n* = 28). Means are shown in each bar with the error bars of the standard error. *** indicates *p* < 0.0005. ALN, ad libitum schedule with non-sweet beverage. ALS, ad libitum schedule with sweet beverage. PRN, progressive ratio schedule with non-sweet beverage. PRS, progressive ratio schedule with sweet beverage.

In patients, CumPres for PRS was no different from the other conditions (*p* > 0.05) (Figure 6). However, in controls, CumPres for PRS was higher than ALN (by 91.4 psi ± 23.2, *p* < 0.0001), ALS (by 83.9 psi ± 23.2, *p* = 0.0003), and PRN (by 81.0 psi ± 23.2, *p* = 0.0005) (Figure 6).

#### 3.2.4. Differences between Patients and Controls for PRS at Both Visits (E3, E6–E8)

**Figure 7:**
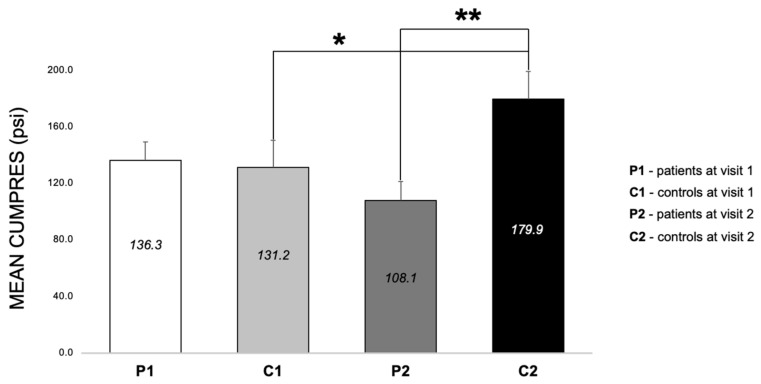
Mean cumulative pressure (MEAN CUMPRES) for the progressive ratio schedule with sweet beverage (PRS) for patients (P, *n* = 56) and controls (C, *n* = 28) at each visit (1—baseline, or 2—3-month follow-up). Means are shown in each bar with the error bars of the standard error. * Indicates *p* < 0.05. ** indicates *p* < 0.005.

There were no CumPres differences between patients and controls in visit one (*p* > 0.05); however, in visit two, the CumPres for patients was lower than controls by 71.8 psi ± 23.4 (*p* = 0.002) (Figure 7). Also of note, in controls, from visit one to visit two, there was an increase in CumPres by 48.7 psi ± 23.2 (*p* = 0.04) (Figure 7).

#### 3.2.5. Specific Interactions

Of all class comparisons across groups (patients vs. controls), visits (1 vs. 2), and conditions (ALN vs. ALS vs. PRN vs. PRS), there were no group × visit interactions; however, there were three group × condition interactions only observed in controls. The visit difference (visit 1–visit 2) of CumPres for PRS (−48.7 psi ± 23.1) was always greater than the visit differences of CumPres for ALN (−2.8 psi ± 23.1), ALS (−6.7 psi ± 23.1), and PRN (−28.1 psi ± 23.1). See Supplementary Table S1. Additionally, there was a significant difference between the PRS and PRN for the controls that was higher than that of patients at visits one and two. At the baseline, this interaction (patient PRS-PRN difference subtracted from control PRS-PRN difference) was 65.6 psi ± 27.3 SE and significant (t_574_ = 2.4, *p* = 0.0167). At follow-up (3 months), the same interaction was 89.0 psi ± 27.3 SE and was even more significant (t_574_ = 3.26, *p* = 0.0012).

### 3.3. ANOVA Model 2—Differences across Visits and Conditions (Schedule × Beverage) in Patients

#### 3.3.1. Overall Model

The overall model for the second ANOVA conducted, which included responses as the dependent variable, and visits (1, 2, and 3) and conditions (beverage × schedule) as the independent variables, was not significant (F-statistic = 0.88, R^2^ = 0.02, Coefficient of Variation = 98.7, *p* = 0.56). The visit and condition effects, and the visit × condition interaction, were not significant (*p* > 0.05, Table 3). Differences and interactions presented in subsequent subsections of 3.3 are derived from this model.

#### 3.3.2. Differences across Visits for Each Condition in Patients (E3 and E4)

**Figure 8:**
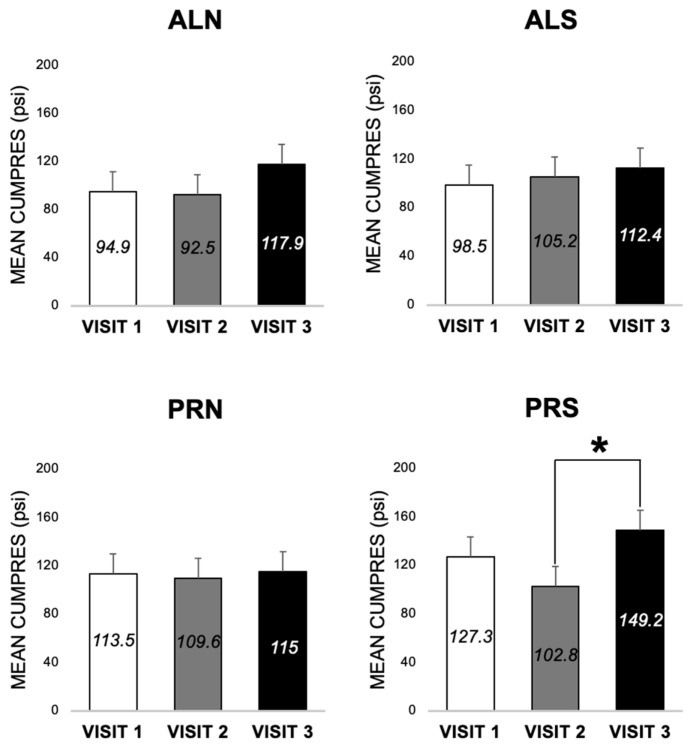
Mean cumulative pressure (MEAN CUMPRES) across three visits for each of the four conditions (schedule × beverage), for patients (*n* = 44). Means are shown in each bar with the error bars of the standard error. * indicates *p* < 0.05. ALN, ad libitum schedule with non-sweet beverage. ALS, ad libitum schedule with sweet beverage. PRN, progressive ratio schedule with non-sweet beverage. PRS, progressive ratio schedule with sweet beverage.

CumPres for ALN, ALS, and PRN, across three visits, did not differ from each other (*p* > 0.05) (Figure 8). CumPres for PRS showed a modest non-significant decrease from visit one (baseline) to visit two (3-month follow-up) by 28.2 psi ± 15.8, *p* = 0.07 (Figure 7). At visit three (24-month follow-up), there was an increase in CumPres from visit two by 46.4 psi ± 20.6, *p* = 0.02) (Figure 8). There was no difference between CumPres at visits one and three (*p* < 0.05), showing a return to baseline (Figure 8).

#### 3.3.3. Differences across Conditions at 24-Month Follow-Up in Patients (E1, E2)

**Figure 9:**
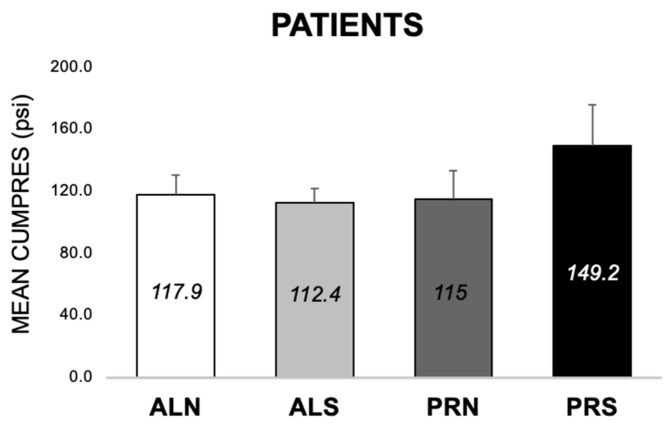
Mean cumulative pressure (MEAN CUMPRES) across four conditions (schedule × beverage) at the 24-month follow-up visit for patients (*n* = 44). Means are shown in each bar with the error bars of the standard error. No means were significantly different from each other (*p* > 0.05). ALN, ad libitum schedule with non-sweet beverage. ALS, ad libitum schedule with sweet beverage. PRN, progressive ratio schedule with non-sweet beverage. PRS, progressive ratio schedule with sweet beverage.

In patients at 24-month follow-up, CumPres for PRS was no different from the other conditions (*p* > 0.05) (Figure 9).

### 3.4. Linear Regressions of Cumulative Pressure (E5 and E9)

#### 3.4.1. Linear Regressions of Cumulative Pressure at Follow-Up from Baseline in Controls (E9)

**Figure 10:**
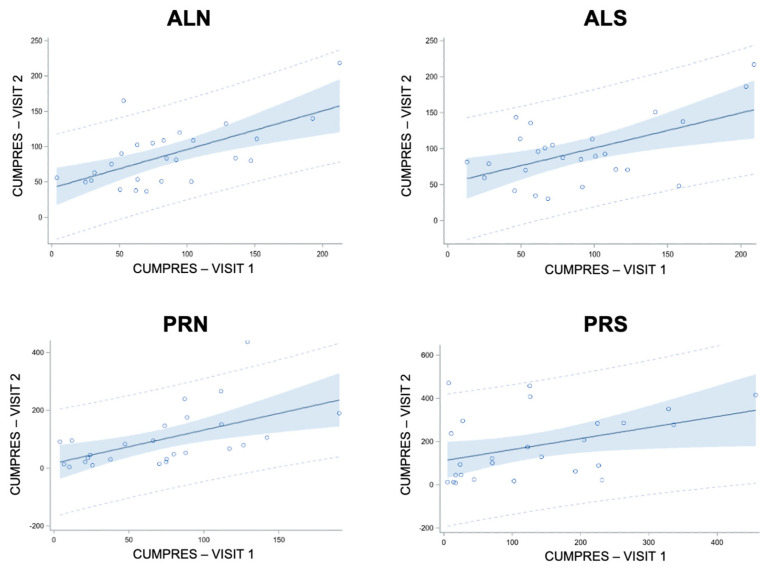
Linear regressions of cumulative pressure (CUMPRES, psi) at visit 2 from visit 1 across conditions (schedule × beverage) for controls (*n* = 28). Statistics for each condition are presented in Table 4. Each circle represents a single participant. The solid line represents the regression line and the dotted lines represent the 95% prediction interval. ALN, ad libitum schedule with non-sweet beverage. ALS, ad libitum schedule with sweet beverage. PRN, progressive ratio schedule with non-sweet beverage. PRS, progressive ratio schedule with sweet beverage.

For this section, all regression plots are in Figure 10 and all regression statistics are in Table 4.

While there was an increase in CumPres for PRS from visit one to visit two in controls (3.2.5), linear regressions of CumPres at visit two from visit one were significant and positive across conditions (*p*’s < 0.04). The intercepts were significant for ALN (*p* = 0.004), ALS (*p* = 0.002), and PRS (*p* = 0.01) but not for PRN (*p* = 0.56)

#### 3.4.2. Linear Regressions of Cumulative Pressure at Follow-Up from Baseline in Patients (E5)

For this section, all regression statistics are in Table 5.

The regression of CumPres at a 3-month follow-up (visit 2) from the baseline (visit 1) (Supplementary Figure S1) was significant for PRS (*p* = 0.01) but not for ALN, ALS, or PRN (*p* > 0.05). The regression of CumPres at a 24-month follow-up (visit 2) from the 3-month follow-up (visit 2) (Supplementary Figure S2) was significant for ALN (*p* = 0.03), ALS (*p* < 0.0001), and PRS (*p* = 0.03), but not for PRN (*p* = 0.09). The regression of CumPres at the 24-month follow-up (visit 2) from visit one (Supplementary Figure S3) was significant for ALS (*p* = 0.01), PRN (*p* = 0.005), and PRS (*p* = 0.01), but not for ALN (*p* = 0.37). All intercepts were significant (*p* < 0.05) except for CumPres of PRS for the regression of visit three from visit one (*p* = 0.07).

## 4. Discussion

### 4.1. Overall Findings

The present study demonstrates that the sipometer is an effective tool for the evaluation of motivation to consume sweet beverages after bariatric surgery. Hogenkamp et al. found that the sipometer is useful for measuring the motivation and reward value of beverages in a small sample of ‘healthy-weight’ humans and is analogous to measures used in animal studies [10]. Their manipulations were deprivation states, sweetness intensity, and sleep restriction [10]. The present study built upon that work by adding BMI and bariatric surgery as manipulations, and recruiting a larger sample to validate this instrument further for use in bariatric surgery patients. The differences in response, as expected [10], were only demonstrated with the progressive ratio schedule when work was required for additional aliquots, specifically of the sweet beverage (PRS).

### 4.2. Specific Findings

#### 4.2.1. Condition Differences (E1 and E2)

We expected that PRS responses would be higher than ALS responses (E1). We found the PRS to be higher than the ALS in patients (significantly at baseline, no different at visit 2, and almost significant at visit 3) and controls (almost significantly at baseline and significantly at visit 2). We also expected that PRN and ALN responses would not differ (E2), and this was true for patients and controls across all study visits. It is important to note that participants did not work as much for the non-sweetened beverage as for the sweetened; thus, the non-sweetened beverage served as a control for generalized responding, and the reinforcement was the sweet taste and not just the beverage itself. The most important finding for conditions was that at 2 years following surgery, the motivation for sweet tastes (PRS), but not for non-sweet tastes (PRN), which decreased at 3 months, not only recovers but actually exceeds responsiveness at 3 months (as shown in Figure 8). For future studies of motivated behavior for sweet taste, the PRS condition is the most likely indicator of responsiveness.

#### 4.2.2. Visit and Surgery Differences (E3 and E4)

We expected that PRS responses would decrease after surgery in patients and that PRN and both AL conditions did not change after surgery (E3 and E4). We found that there was a drop in PRS responses in patients 3 months after surgery; however, the motivation for sweet tastes increased at 24 months, showing a return to pre-surgery baseline levels. This pattern tracks the immediate decrease in sweet-liking [9,44,46] and sweet-beverage intake [16,25,27,47] after bariatric surgery and a subsequent increase in sweet-liking [48] and sweet-beverage intake [49] during long-term intake in post-op bariatric surgery patients.

#### 4.2.3. Group Differences (E6 and E7)

We expected the controls’ PRS responses to be similar at the baseline (E6) and patients’ PRS responses to be lower than the controls’ at the follow-up (E7). We found both of our expected outcomes to be accurate. Patients worked less for the sweet beverage compared to controls at 3 months. However, the controls also increased their PRS response at visit two, which could explain the significance of this group difference after surgery. Regardless, patients’ PRS responses were lower than controls’ on average.

#### 4.2.4. Control Differences (E8)

We expected that responses on both AL and PR schedules for sweet and non-sweet liquids would not change over time in controls (E8). Unexpectedly, the controls, who had greater variation in their cumulative pressures under the PR schedule compared to patients, significantly increased their PRS responses at visit two, which shows that motivation to consume sweet items may vary. This result could also indicate some effect of learning and familiarity with the task. Control responses for ad libitum (no work needed) and progressive ratio cumulative pressures for a non-preferred, non-sweet item were stable and were good controls for this experiment. Overall, the pattern of responsiveness was well-preserved in controls over time; that is, PRS response was consistently higher than that of PRN, ALN, and ALS conditions in controls. More work should be performed on replication across trials in the future to confidently prove test–retest reliability of PRS response.

#### 4.2.5. Regressions across Visits (E5 and E9)

We expected that responses on both schedules for sweet and non-sweet liquids would correlate over time in controls (E9), and this was confirmed. All correlations were significant but had slopes less than one, with below-one slopes across all conditions. Nevertheless, responses between the two visits were strongly associated with one another, which demonstrates that the procedure was relatively reliable and reproducible. The increase in the intercept only under PRS indicates that some aspect, possibly increased familiarity with the procedure, increased responsiveness independently of the individuals’ responses. The fact that this increase only occurred for the sweet but not the non-sweet taste indicates specificity for a known reinforcing a sweet-taste property of the beverage.

We expected that patients’ responses would correlate across visits for each condition, more strongly for non-sweet tastes and AL conditions than for PRS, whose slope across visits would be reduced compared to the other conditions (E5). This expected outcome was only partially confirmed. Patients’ lack of persistence across conditions between visits one and two, with the exception of the PRS, is likely the result of the generally high level of variability. The PRS slope was significant but low (0.31 psi/psi), which indicates that the sweet taste responsiveness was reduced after surgery and that visit two responses remained proportional but lower than visit one. Interestingly, the slopes of all conditions, when regressing visit three from visit two, were higher than the slopes for regressions of visit two from visit one. They were also significant, except for PRN, which shows that there was a stronger association of post-op motivation over time. The significant correlations between visit three and visit one, and higher slopes for PRS than the other three conditions, are consistent with the revived motivation for sweet taste at 2 years, which is in proportion to the motivation before surgery. There was more variability in the intercept at visit three for PRS; hence, it was not significant, although it was not different from the intercepts from the regressions of visit three from two (83.39 psi) or of visit two from one (63.40 psi).

### 4.3. Advantages

One important advantage of using the sipometer is that it is easy to use for both the research assistant and the study participant. It is a safe and clean laboratory method for measuring the motivation to consume sweet beverages and can be used for liquid meals as well. The present study further shows how important it is for post-bariatric surgery studies; at 3 months post-op, many patients are advised to limit their solid food intake [50], and the sham-feeding conducted in this study allowed us to measure the motivation and reward value of sweet non-caloric beverages while avoiding the post-ingestive effects that put our patients at ease. Patients reported no discomfort or hesitance after completing this task.

Another advantage is that the procedure collects and outputs data in a way that eating and drinking microstructures [51] can be easily studied. While our main measure of motivation was cumulative pressure, duration (time during and between sipping), intake, changes in pressure during bursts, burst size, frequency of bursts, and other parameters can be examined.

### 4.4. Limitations

One limitation of the present study is that while we were able to measure the motivation to consume sweet beverages, we do not know how this relates to the motivation to consume sweet solid foods and how these two relate to the actual consumption of sweet beverages and food items. We also did not test how liking drives motivation over time, and we did not compare nutritive and nonnutritive beverages in this task. We also do not know how important the flavor was in driving the response because we did not use a non-flavored control, which could have resulted in very little response. Since our main goal was to demonstrate that sweet taste was motivating, we followed procedures used by others [52] by simply adding flavor to the sweet taste that would presumably facilitate responding.

Another limitation was our time points. Three months post-operation provided us with an interesting snapshot of patients’ drinking microstructures during drastic short-term weight loss, but it may be a time point that is too soon to measure motivational changes [51]. To remedy that, ideally, we would have multiple visits between 3 and 24 months. Having multiple points, at least four, could allow for the examination of motivation curves rather than differences. Furthermore, additional control visits could help elucidate how controls respond over a longer period of time.

### 4.5. Future Directions

Because we have illustrated that the sipometer is measuring motivation, it would be beneficial, as mentioned, to see how motivation is associated with liking, sweet taste acuity [17], and actual intake [53] as they change post-operatively. Further, we demonstrated that there was a rebound in motivation in patients, and it would be valuable to see if this pattern tracks the often-discussed rebound in body weight loss in bariatric surgery patients [54]. We also do not know how motivation is related to other possibly informative ‘emotional’ variables (such as disinhibition, dietary restraints [55], binge eating [56], emotional eating [57,58], and depression [57,59]) as these variables may provide context to a patient’s motivation to consume sweet and their post-operative outcomes. It would also be advantageous to test the baseline and early post-operative motivation as predictors of long-term post-operative intake and weight loss. Virtual reports of sweet item consumption under an ‘unlimited’ theoretical eating scenario predicted post-operative weight loss in bariatric patients [60], and motivation to consume sweet items may be an explanatory variable or mediator to this relationship and should be explored as well. These future findings could inform additional pre-surgical interventions that address the motivation to consume sweet things if a higher baseline motivation to consume sweet things either predicts worse post-operative outcomes or indicates who will benefit most from the surgery.

The sipometer also offers a novel method for the evaluation of the motivation to consume other fluids, including nutritive and nonnutritive beverages with varying proportions of macronutrients and concentrations, sports drinks, soft drinks, and caffeinated drinks (tea and coffee) in situations where they are both swallowed or expectorated to remove post-ingestion effects. Given the previously noted findings on preferences at various concentrations, it would be important to repeat these studies with stronger concentrations. It is expected that there will be a greater reduction with a 1 M sucrose equivalent solution at 3 months post-surgery and a greater rebound at 2 years. The paradigm we used and the device in which it was employed, therefore, have the potential for further investigation in which nutritive and LCSs could be compared.

## 5. Conclusions

In conclusion, the sipometer has demonstrated relative reliability and sensitivity for the measure of motivation to consume sweet beverages in bariatric surgery patients. This instrument could help us understand changes in energy intake after bariatric surgery.

## Figures and Tables

**Table 1:** Participant demographics ^1^.

Demographics	Patients *n =* 56	Controls *n =* 28
Age, y	34.9 ± 1.3	34.0 ± 2.1
Sex	89% F/11% M	79% F/21% M
Race	55% B/45% NB	61% B/39% NB
Ethnicity	64% H/36% NH	68% H/32% NH
Surgery type	38% R/62% V	-
Weight, kg	121.7 ± 2.6 ^2^	59.8 ± 1.3
BMI, kg/m^2^	44.8 ± 0.8 ^2^	21.8 ± 0.32
Weight loss, 3 months, kg	20.7 ± 0.7	−0.4 ± 0.4
Weight loss, 3 months, %	17.1 ± 0.5	−0.7 ± 0.6
Weight loss, 24 months, kg ^3^	34.4 ± 2.1	-
Weight loss, 24 months, % ^3^	28.1 ± 1.6	-

^1^ This table contains participant demographics for patients and controls at baseline. Values are means ± SEs unless otherwise indicated. F, female; M, male; B, Black; NB, non-Black; H, Hispanic; NH, non-Hispanic; R, Roux-en-Y gastric bypass; V, vertical sleeve gastrectomy. ^2^ Significant group differences (*t*-test, *p* < 0.0001). ^3^ Weight loss at 24 months has an *n* of 44 due to the loss of 11 patients to follow-up and 1 patient due to incomplete data.

**Table 2:** Overall effects and interactions ^1^.

Effect	df	F-Value	*p*-Value
Group	1	0.71	0.40
Visit	1	0.05	0.83
Condition	3	2.02	0.16
Group × Visit	1	10.90	<0.0001
Group × Condition	3	4.17	0.006
Visit × Condition	3	0.28	0.84
Group × Visit × Condition	3	0.40	0.75

^1^ This table contains the Type III effects and interactions for the first analysis of variance conducted. The model included response as the dependent variable and group (patients, *n* = 56, and controls, *n =* 28), visit (1 or 2), and condition as independent variables.

**Table 3:** Overall effects and interactions ^1^.

Effect	df	F-Value	*p*-Value
Visit	2	2.22	0.11
Condition	3	1.68	0.17
Visit × Condition	6	0.50	0.81

^1^ This table contains the Type III effects and visit × condition interaction for the second analysis of variance conducted in patients (*n =* 44). The model included response as the dependent variable and visit (1, 2, or 3), and condition as independent variables.

**Table 4:** Linear regression statistics for controls ^1^.

Cond	F-Value	Rsq	Slope ± SE	Slope P	Intercept ± SE	Intercept P
ALN	16.79	0.41	0.55 ± 0.13	0.0004	41.72 ± 13.21	0.004
ALS	10.59	0.31	0.49 ± 0.15	0.003	52.41 ± 15.39	0.002
PRN	11.03	0.31	1.15 ± 0.35	0.003	17.48 ± 29.53	0.56
PRS	4.82	0.17	0.51 ± 0.23	0.04	112.96 ± 41.35	0.01

^1^ This table contains the regression statistics for linear regressions of cumulative pressure at visit 2 from visit 1 across conditions (cond: schedule × beverage) in controls (*n* = 28). *p*-values held to α = 0.05. ALN, ad libitum schedule with non-sweet beverage. ALS, ad libitum schedule with sweet beverage. PRN, progressive ratio schedule with non-sweet beverage. PRS, progressive ratio schedule with sweet beverage.

**Table 5:** Linear regression statistics for patients ^1^.

Dep	Indep	Cond	F-Value	Rsq	Slope ± SE	Slope P	Intercept ± SE	Intercept P
V2	V1	ALN	0.00	0.00	−0.005 ± 0.19	0.98	92.90 ± 20.44	<0.0001
		ALS	0.23	0.005	0.09 ± 0.19	0.64	96.37 ± 21.34	<0.0001
		PRN	0.00	0.00	0.004 ± 0.12	0.97	109.14 ± 25.63	0.0001
		PRS	6.87	0.14	0.31 ± 0.12	0.01	63.40 ± 19.86	0.003
V3	V2	ALN	4.92	0.10	0.39 ± 0.18	0.03	81.59 ± 20.25	0.0002
		ALS	21.61	0.34	0.53 ± 0.11	<0.0001	56.21 ± 14.48	0.0004
		PRN	2.99	0.07	0.22 ± 0.13	0.09	90.60 ± 22.79	0.0003
		PRS	4.89	0.10	0.63 ± 0.29	0.03	83.39 ± 39.10	0.04
V3	V1	ALN	0.83	0.02	0.20 ± 0.22	0.37	98.63 ± 24.51	0.0002
		ALS	7.29	0.15	0.43 ± 0.16	0.01	70.09 ± 18.09	0.0004
		PRN	8.93	0.18	0.29 ± 0.10	0.005	82.16 ± 20.09	0.0002
		PRS	6.99	0.14	0.61 ± 0.23	0.01	71.67 ± 38.73	0.07

^1^ This table contains the regression statistics for linear regressions of cumulative pressure (psi) at visit 2 (V2) from visit 1 (V1), visit 3 (V3) from V2, and V3 from V1, across conditions (schedule × beverage) in patients (*n* = 44). The *p*-values were held to α = 0.05. Dep, dependent variable. Indep, independent variable. ALN, ad libitum schedule with non-sweet beverage. ALS, ad libitum schedule with sweet beverage. PRN, progressive ratio schedule with non-sweet beverage. PRS, progressive ratio schedule with sweet beverage.

## Data Availability

Data and SAS code are available upon request to the corresponding author.

## Supplementary Materials

The following supporting information can be downloaded at: https://www.mdpi.com/article/10.3390/nu16173001/s1, Figure S1: Linear regressions of cumulative pressure (CUMPRES, psi) at visit 2 from visit 1 across conditions (schedule × beverage) for patients (*n* = 44). Statistics for each condition are presented in the text (Table 5). ALN, ad libitum schedule with non-sweet beverage. ALS, ad libitum schedule with sweet beverage. PRN, progressive ratio schedule with non-sweet beverage. PRS, progressive ratio schedule with sweet beverage; Figure S2: Linear regressions of cumulative pressure (CUMPRES, psi) at visit 3 from visit 2 across conditions (schedule × beverage) for patients (*n* = 44). Statistics for each condition are presented in the text (Table 5). ALN, ad libitum schedule with non-sweet beverage. ALS, ad libitum schedule with sweet beverage. PRN, progressive ratio schedule with non-sweet beverage. PRS, progressive ratio schedule with sweet beverage; Figure S3: Linear regressions of cumulative pressure (CUMPRES, psi) at visit 3 from visit 1 across conditions (schedule × beverage) for patients (*n* = 44). Statistics for each condition are presented in the text (Table 5). ALN, ad libitum schedule with non-sweet beverage. ALS, ad libitum schedule with sweet beverage. PRN, progressive ratio schedule with non-sweet beverage. PRS, progressive ratio schedule with sweet beverage; Table S1: Means and interactions.
