# Supplementary material for: Responsiveness and Reliability of a Sipping Device to Measure Motivation in Normal-Weight Individuals and Bariatric Surgery Patients
Source: Nutrients. 2024 Sep 5;16(17):3001. doi: 10.3390/nu16173001 (PMC11396939; doi:10.3390/nu16173001)
Supplement: Supplementary file 1 [file nutrients-16-03001-s001.zip › nutrients-3110475-supplementary.pdf]

SUPPLEMENTAL TABLES

Supplementary Table S1. Means and interactions[1]

Effect	GROUP	COND	VISIT	_GROUP	_COND	_VISIT	Estimate	Standard Error	t Value	Pr > |t|	Tukey P
GROUP	C			P			-1.83	14.32	-0.13	0.8986	0.8986
COND		ALN			ALS		-7.75	9.91	-0.78	0.4342	0.8624
COND		ALN			PRN		-7.38	9.91	-0.74	0.4568	0.8789
COND		ALN			PRS		-48.96	9.91	-4.94	<.0001	<.0001
COND		ALS			PRN		0.38	9.91	0.04	0.9698	1.0000
COND		ALS			PRS		-41.21	9.91	-4.16	<.0001	0.0002
COND		PRN			PRS		-41.58	9.91	-4.20	<.0001	0.0002
GROUP*VISIT	C		1	C		2	-21.58	11.58	-1.86	0.0628	0.2448
GROUP*VISIT	C		1	P		1	-17.74	15.94	-1.11	0.2661	0.6815
GROUP*VISIT	C		1	P		2	-7.49	15.94	-0.47	0.6386	0.9656
GROUP*VISIT	C		2	P		1	3.84	15.94	0.24	0.8098	0.9951
GROUP*VISIT	C		2	P		2	14.09	15.94	0.88	0.3770	0.8131
GROUP*VISIT	P		1	P		2	10.25	7.89	1.30	0.1942	0.5635
GROUP*COND	C	ALN		C	ALS		-5.46	16.37	-0.33	0.7388	1.0000
GROUP*COND	C	ALN		C	PRN		2.30	16.37	0.14	0.8885	1.0000
GROUP*COND	C	ALN		C	PRS		-68.41	16.37	-4.18	<.0001	0.0009
GROUP*COND	C	ALN		P	ALN		-5.57	18.76	-0.30	0.7667	1.0000
GROUP*COND	C	ALN		P	ALS		-15.61	18.76	-0.83	0.4058	0.9912
GROUP*COND	C	ALN		P	PRN		-22.62	18.76	-1.21	0.2285	0.9302
GROUP*COND	C	ALN		P	PRS		-35.08	18.76	-1.87	0.0621	0.5728
GROUP*COND	C	ALS		C	PRN		7.76	16.37	0.47	0.6358	0.9998
GROUP*COND	C	ALS		C	PRS		-62.95	16.37	-3.84	0.0001	0.0034
GROUP*COND	C	ALS		P	ALN		-0.11	18.76	-0.01	0.9954	1.0000
GROUP*COND	C	ALS		P	ALS		-10.15	18.76	-0.54	0.5888	0.9994
GROUP*COND	C	ALS		P	PRN		-17.16	18.76	-0.91	0.3609	0.9847
GROUP*COND	C	ALS		P	PRS		-29.61	18.76	-1.58	0.1151	0.7634
GROUP*COND	C	PRN		C	PRS		-70.71	16.37	-4.32	<.0001	0.0005
GROUP*COND	C	PRN		P	ALN		-7.87	18.76	-0.42	0.6752	0.9999
GROUP*COND	C	PRN		P	ALS		-17.91	18.76	-0.95	0.3403	0.9803
GROUP*COND	C	PRN		P	PRN		-24.92	18.76	-1.33	0.1848	0.8881
GROUP*COND	C	PRN		P	PRS		-37.37	18.76	-1.99	0.0469	0.4885
GROUP*COND	C	PRS		P	ALN		62.84	18.76	3.35	0.0009	0.0195
GROUP*COND	C	PRS		P	ALS		52.80	18.76	2.81	0.0051	0.0936

GROUP*COND	C	PRS		P	PRN		45.79	18.76	2.44	0.0150	0.2241
GROUP*COND	C	PRS		P	PRS		33.34	18.76	1.78	0.0762	0.6363
GROUP*COND	P	ALN		P	ALS		-10.04	11.16	-0.90	0.3685	0.9860
GROUP*COND	P	ALN		P	PRN		-17.05	11.16	-1.53	0.1270	0.7919
GROUP*COND	P	ALN		P	PRS		-29.50	11.16	-2.64	0.0084	0.1424
GROUP*COND	P	ALS		P	PRN		-7.01	11.16	-0.63	0.5301	0.9985
GROUP*COND	P	ALS		P	PRS		-19.46	11.16	-1.74	0.0816	0.6578
GROUP*COND	P	PRN		P	PRS		-12.45	11.16	-1.12	0.2647	0.9532
VISIT			1			2	-5.66	7.00	-0.81	0.4191	0.4191
GROUP*VISIT*COND	C	ALN	1	C	ALS	1	-3.49	23.15	-0.15	0.8802	1.0000
GROUP*VISIT*COND	C	ALN	1	C	PRN	1	14.95	23.15	0.65	0.5187	1.0000
GROUP*VISIT*COND	C	ALN	1	C	PRS	1	-45.47	23.15	-1.96	0.0501	0.8442
GROUP*VISIT*COND	C	ALN	1	C	ALN	2	-2.79	23.15	-0.12	0.9041	1.0000
GROUP*VISIT*COND	C	ALN	1	C	ALS	2	-10.23	23.15	-0.44	0.6589	1.0000
GROUP*VISIT*COND	C	ALN	1	C	PRN	2	-13.15	23.15	-0.57	0.5702	1.0000
GROUP*VISIT*COND	C	ALN	1	C	PRS	2	-94.15	23.15	-4.07	<.0001	0.0055
GROUP*VISIT*COND	C	ALN	1	P	ALN	1	-9.91	23.42	-0.42	0.6723	1.0000
GROUP*VISIT*COND	C	ALN	1	P	ALS	1	-16.32	23.42	-0.70	0.4862	1.0000
GROUP*VISIT*COND	C	ALN	1	P	PRN	1	-28.18	23.42	-1.20	0.2294	0.9982
GROUP*VISIT*COND	C	ALN	1	P	PRS	1	-50.56	23.42	-2.16	0.0313	0.7254
GROUP*VISIT*COND	C	ALN	1	P	ALN	2	-4.02	23.42	-0.17	0.8637	1.0000
GROUP*VISIT*COND	C	ALN	1	P	ALS	2	-17.70	23.42	-0.76	0.4501	1.0000
GROUP*VISIT*COND	C	ALN	1	P	PRN	2	-19.85	23.42	-0.85	0.3969	1.0000
GROUP*VISIT*COND	C	ALN	1	P	PRS	2	-22.38	23.42	-0.96	0.3396	0.9999
GROUP*VISIT*COND	C	ALS	1	C	PRN	1	18.44	23.15	0.80	0.4260	1.0000
GROUP*VISIT*COND	C	ALS	1	C	PRS	1	-41.97	23.15	-1.81	0.0704	0.9111
GROUP*VISIT*COND	C	ALS	1	C	ALN	2	0.70	23.15	0.03	0.9759	1.0000
GROUP*VISIT*COND	C	ALS	1	C	ALS	2	-6.74	23.15	-0.29	0.7712	1.0000
GROUP*VISIT*COND	C	ALS	1	C	PRN	2	-9.66	23.15	-0.42	0.6766	1.0000
GROUP*VISIT*COND	C	ALS	1	C	PRS	2	-90.66	23.15	-3.92	0.0001	0.0098
GROUP*VISIT*COND	C	ALS	1	P	ALN	1	-6.42	23.42	-0.27	0.7840	1.0000
GROUP*VISIT*COND	C	ALS	1	P	ALS	1	-12.83	23.42	-0.55	0.5841	1.0000
GROUP*VISIT*COND	C	ALS	1	P	PRN	1	-24.69	23.42	-1.05	0.2922	0.9996
GROUP*VISIT*COND	C	ALS	1	P	PRS	1	-47.07	23.42	-2.01	0.0449	0.8189
GROUP*VISIT*COND	C	ALS	1	P	ALN	2	-0.53	23.42	-0.02	0.9819	1.0000
GROUP*VISIT*COND	C	ALS	1	P	ALS	2	-14.21	23.42	-0.61	0.5443	1.0000
GROUP*VISIT*COND	C	ALS	1	P	PRN	2	-16.36	23.42	-0.70	0.4850	1.0000
GROUP*VISIT*COND	C	ALS	1	P	PRS	2	-18.89	23.42	-0.81	0.4202	1.0000

GROUP*VISIT*COND	C	PRN	1	C	PRS	1	-60.42	23.15	-2.61	0.0093	0.3941
GROUP*VISIT*COND	C	PRN	1	C	ALN	2	-17.74	23.15	-0.77	0.4438	1.0000
GROUP*VISIT*COND	C	PRN	1	C	ALS	2	-25.18	23.15	-1.09	0.2773	0.9994
GROUP*VISIT*COND	C	PRN	1	C	PRN	2	-28.11	23.15	-1.21	0.2253	0.9980
GROUP*VISIT*COND	C	PRN	1	C	PRS	2	-109.11	23.15	-4.71	<.0001	0.0003
GROUP*VISIT*COND	C	PRN	1	P	ALN	1	-24.87	23.42	-1.06	0.2888	0.9996
GROUP*VISIT*COND	C	PRN	1	P	ALS	1	-31.27	23.42	-1.34	0.1823	0.9944
GROUP*VISIT*COND	C	PRN	1	P	PRN	1	-43.13	23.42	-1.84	0.0660	0.9000
GROUP*VISIT*COND	C	PRN	1	P	PRS	1	-65.51	23.42	-2.80	0.0053	0.2734
GROUP*VISIT*COND	C	PRN	1	P	ALN	2	-18.98	23.42	-0.81	0.4181	1.0000
GROUP*VISIT*COND	C	PRN	1	P	ALS	2	-32.65	23.42	-1.39	0.1638	0.9913
GROUP*VISIT*COND	C	PRN	1	P	PRN	2	-34.81	23.42	-1.49	0.1377	0.9838
GROUP*VISIT*COND	C	PRN	1	P	PRS	2	-37.34	23.42	-1.59	0.1114	0.9690
GROUP*VISIT*COND	C	PRS	1	C	ALN	2	42.67	23.15	1.84	0.0659	0.8996
GROUP*VISIT*COND	C	PRS	1	C	ALS	2	35.24	23.15	1.52	0.1286	0.9797
GROUP*VISIT*COND	C	PRS	1	C	PRN	2	32.31	23.15	1.40	0.1634	0.9913
GROUP*VISIT*COND	C	PRS	1	C	PRS	2	-48.69	23.15	-2.10	0.0359	0.7628
GROUP*VISIT*COND	C	PRS	1	P	ALN	1	35.55	23.42	1.52	0.1295	0.9802
GROUP*VISIT*COND	C	PRS	1	P	ALS	1	29.15	23.42	1.24	0.2138	0.9974
GROUP*VISIT*COND	C	PRS	1	P	PRN	1	17.29	23.42	0.74	0.4607	1.0000
GROUP*VISIT*COND	C	PRS	1	P	PRS	1	-5.10	23.42	-0.22	0.8278	1.0000
GROUP*VISIT*COND	C	PRS	1	P	ALN	2	41.44	23.42	1.77	0.0773	0.9260
GROUP*VISIT*COND	C	PRS	1	P	ALS	2	27.77	23.42	1.19	0.2362	0.9985
GROUP*VISIT*COND	C	PRS	1	P	PRN	2	25.61	23.42	1.09	0.2746	0.9994
GROUP*VISIT*COND	C	PRS	1	P	PRS	2	23.08	23.42	0.99	0.3247	0.9998
GROUP*VISIT*COND	C	ALN	2	C	ALS	2	-7.43	23.15	-0.32	0.7483	1.0000
GROUP*VISIT*COND	C	ALN	2	C	PRN	2	-10.36	23.15	-0.45	0.6547	1.0000
GROUP*VISIT*COND	C	ALN	2	C	PRS	2	-91.36	23.15	-3.95	<.0001	0.0087
GROUP*VISIT*COND	C	ALN	2	P	ALN	1	-7.12	23.42	-0.30	0.7612	1.0000
GROUP*VISIT*COND	C	ALN	2	P	ALS	1	-13.53	23.42	-0.58	0.5638	1.0000
GROUP*VISIT*COND	C	ALN	2	P	PRN	1	-25.39	23.42	-1.08	0.2788	0.9995
GROUP*VISIT*COND	C	ALN	2	P	PRS	1	-47.77	23.42	-2.04	0.0418	0.8017
GROUP*VISIT*COND	C	ALN	2	P	ALN	2	-1.23	23.42	-0.05	0.9581	1.0000
GROUP*VISIT*COND	C	ALN	2	P	ALS	2	-14.91	23.42	-0.64	0.5247	1.0000
GROUP*VISIT*COND	C	ALN	2	P	PRN	2	-17.06	23.42	-0.73	0.4665	1.0000
GROUP*VISIT*COND	C	ALN	2	P	PRS	2	-19.59	23.42	-0.84	0.4032	1.0000
GROUP*VISIT*COND	C	ALS	2	C	PRN	2	-2.93	23.15	-0.13	0.8995	1.0000
GROUP*VISIT*COND	C	ALS	2	C	PRS	2	-83.93	23.15	-3.62	0.0003	0.0276

GROUP*VISIT*COND	C	ALS	2	P	ALN	1	0.31	23.42	0.01	0.9893	1.0000
GROUP*VISIT*COND	C	ALS	2	P	ALS	1	-6.09	23.42	-0.26	0.7949	1.0000
GROUP*VISIT*COND	C	ALS	2	P	PRN	1	-17.95	23.42	-0.77	0.4436	1.0000
GROUP*VISIT*COND	C	ALS	2	P	PRS	1	-40.33	23.42	-1.72	0.0855	0.9403
GROUP*VISIT*COND	C	ALS	2	P	ALN	2	6.20	23.42	0.26	0.7912	1.0000
GROUP*VISIT*COND	C	ALS	2	P	ALS	2	-7.47	23.42	-0.32	0.7498	1.0000
GROUP*VISIT*COND	C	ALS	2	P	PRN	2	-9.63	23.42	-0.41	0.6811	1.0000
GROUP*VISIT*COND	C	ALS	2	P	PRS	2	-12.16	23.42	-0.52	0.6039	1.0000
GROUP*VISIT*COND	C	PRN	2	C	PRS	2	-81.00	23.15	-3.50	0.0005	0.0418
GROUP*VISIT*COND	C	PRN	2	P	ALN	1	3.24	23.42	0.14	0.8900	1.0000
GROUP*VISIT*COND	C	PRN	2	P	ALS	1	-3.16	23.42	-0.14	0.8925	1.0000
GROUP*VISIT*COND	C	PRN	2	P	PRN	1	-15.03	23.42	-0.64	0.5214	1.0000
GROUP*VISIT*COND	C	PRN	2	P	PRS	1	-37.41	23.42	-1.60	0.1107	0.9685
GROUP*VISIT*COND	C	PRN	2	P	ALN	2	9.13	23.42	0.39	0.6967	1.0000
GROUP*VISIT*COND	C	PRN	2	P	ALS	2	-4.55	23.42	-0.19	0.8461	1.0000
GROUP*VISIT*COND	C	PRN	2	P	PRN	2	-6.70	23.42	-0.29	0.7748	1.0000
GROUP*VISIT*COND	C	PRN	2	P	PRS	2	-9.23	23.42	-0.39	0.6936	1.0000
GROUP*VISIT*COND	C	PRS	2	P	ALN	1	84.24	23.42	3.60	0.0003	0.0302
GROUP*VISIT*COND	C	PRS	2	P	ALS	1	77.84	23.42	3.32	0.0009	0.0716
GROUP*VISIT*COND	C	PRS	2	P	PRN	1	65.97	23.42	2.82	0.0050	0.2622
GROUP*VISIT*COND	C	PRS	2	P	PRS	1	43.59	23.42	1.86	0.0632	0.8920
GROUP*VISIT*COND	C	PRS	2	P	ALN	2	90.13	23.42	3.85	0.0001	0.0125
GROUP*VISIT*COND	C	PRS	2	P	ALS	2	76.45	23.42	3.26	0.0012	0.0850
GROUP*VISIT*COND	C	PRS	2	P	PRN	2	74.30	23.42	3.17	0.0016	0.1100
GROUP*VISIT*COND	C	PRS	2	P	PRS	2	71.77	23.42	3.06	0.0023	0.1464
GROUP*VISIT*COND	P	ALN	1	P	ALS	1	-6.41	15.78	-0.41	0.6849	1.0000
GROUP*VISIT*COND	P	ALN	1	P	PRN	1	-18.27	15.78	-1.16	0.2474	0.9988
GROUP*VISIT*COND	P	ALN	1	P	PRS	1	-40.65	15.78	-2.58	0.0102	0.4173
GROUP*VISIT*COND	P	ALN	1	P	ALN	2	5.89	15.78	0.37	0.7090	1.0000
GROUP*VISIT*COND	P	ALN	1	P	ALS	2	-7.79	15.78	-0.49	0.6218	1.0000
GROUP*VISIT*COND	P	ALN	1	P	PRN	2	-9.94	15.78	-0.63	0.5288	1.0000
GROUP*VISIT*COND	P	ALN	1	P	PRS	2	-12.47	15.78	-0.79	0.4296	1.0000
GROUP*VISIT*COND	P	ALS	1	P	PRN	1	-11.86	15.78	-0.75	0.4525	1.0000
GROUP*VISIT*COND	P	ALS	1	P	PRS	1	-34.24	15.78	-2.17	0.0304	0.7176
GROUP*VISIT*COND	P	ALS	1	P	ALN	2	12.30	15.78	0.78	0.4361	1.0000
GROUP*VISIT*COND	P	ALS	1	P	ALS	2	-1.38	15.78	-0.09	0.9303	1.0000
GROUP*VISIT*COND	P	ALS	1	P	PRN	2	-3.54	15.78	-0.22	0.8227	1.0000
GROUP*VISIT*COND	P	ALS	1	P	PRS	2	-6.06	15.78	-0.38	0.7008	1.0000

GROUP*VISIT*COND	P	PRN	1	P	PRS	1	-22.38	15.78	-1.42	0.1566	0.9897
GROUP*VISIT*COND	P	PRN	1	P	ALN	2	24.16	15.78	1.53	0.1263	0.9785
GROUP*VISIT*COND	P	PRN	1	P	ALS	2	10.48	15.78	0.66	0.5068	1.0000
GROUP*VISIT*COND	P	PRN	1	P	PRN	2	8.32	15.78	0.53	0.5980	1.0000
GROUP*VISIT*COND	P	PRN	1	P	PRS	2	5.80	15.78	0.37	0.7135	1.0000
GROUP*VISIT*COND	P	PRS	1	P	ALN	2	46.54	15.78	2.95	0.0033	0.1944
GROUP*VISIT*COND	P	PRS	1	P	ALS	2	32.86	15.78	2.08	0.0377	0.7754
GROUP*VISIT*COND	P	PRS	1	P	PRN	2	30.71	15.78	1.95	0.0521	0.8530
GROUP*VISIT*COND	P	PRS	1	P	PRS	2	28.18	15.78	1.79	0.0746	0.9206
GROUP*VISIT*COND	P	ALN	2	P	ALS	2	-13.68	15.78	-0.87	0.3864	1.0000
GROUP*VISIT*COND	P	ALN	2	P	PRN	2	-15.83	15.78	-1.00	0.3160	0.9998
GROUP*VISIT*COND	P	ALN	2	P	PRS	2	-18.36	15.78	-1.16	0.2450	0.9988
GROUP*VISIT*COND	P	ALS	2	P	PRN	2	-2.16	15.78	-0.14	0.8914	1.0000
GROUP*VISIT*COND	P	ALS	2	P	PRS	2	-4.68	15.78	-0.30	0.7667	1.0000
GROUP*VISIT*COND	P	PRN	2	P	PRS	2	-2.53	15.78	-0.16	0.8728	1.0000

[1] This table includes all means and interactions in the ANOVA model that we employed. The model was CUMPRESSOFF = GROUP | VISIT | COND, where CUMPRESSOF was the cumulative pressure (psi), GROUP was the group class variable for patients and controls, VISIT was the visit class variable for visit 1 and 2, and COND was the condition class variable for ALN, ALS, PRN, or PRS. Degrees of freedom = 560.

SUPPLEMENTAL FIGURES

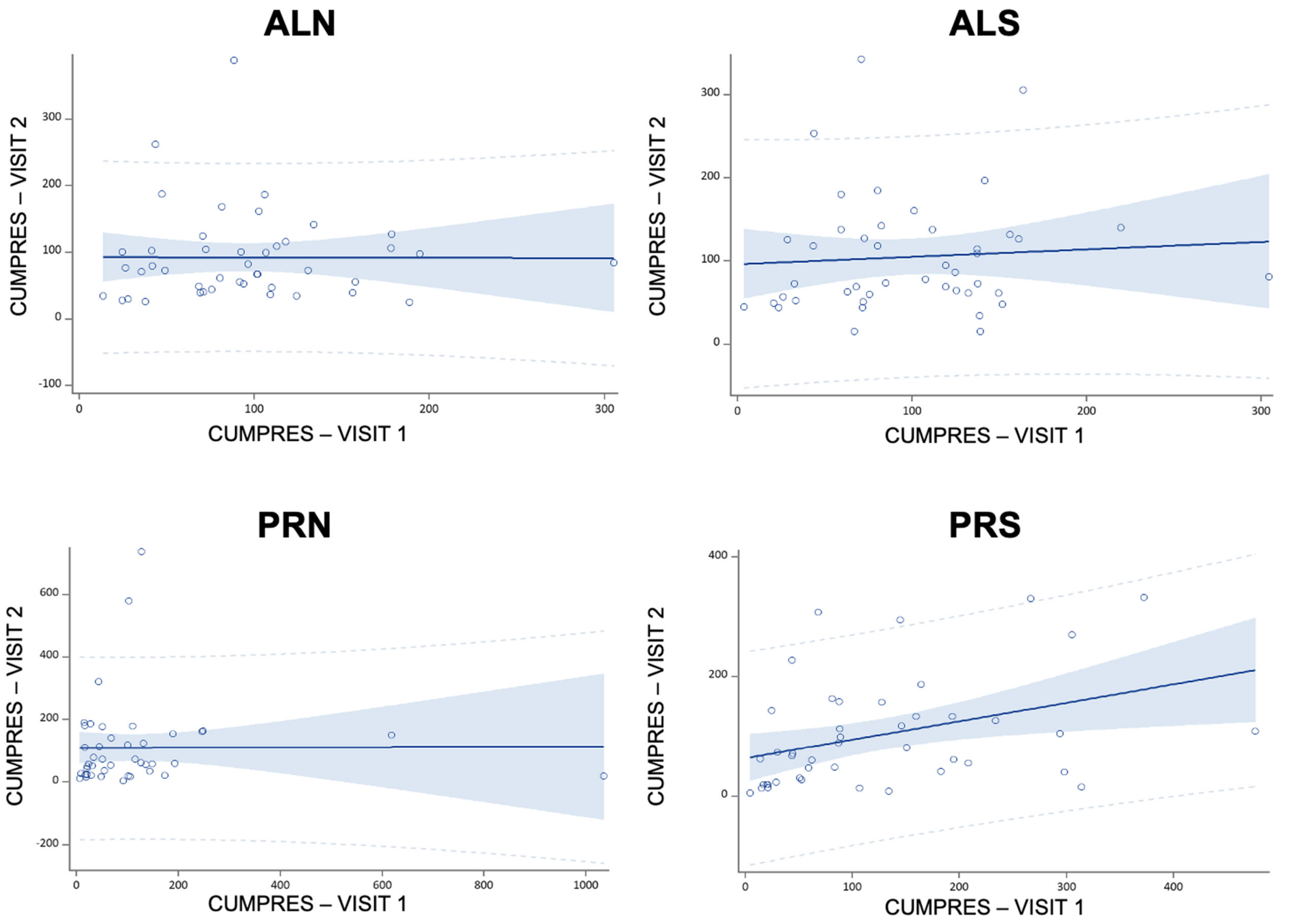

Supplementary Figure S1. Linear regressions of cumulative pressure (CUMPRES, psi) at visit 2 from visit 1 across conditions (schedule x beverage) for patients (n = 44). Statistics for each condition are presented in the text (Table 5). Each circle represents a single participant. The solid line represents the regression line and the dotted lines represent the 95% prediction interval. ALN, ad libitum schedule with non-sweet beverage. ALS, ad libitum schedule with sweet beverage. PRN, progressive ratio schedule with non-sweet beverage. PRS, progressive ratio schedule with sweet beverage.

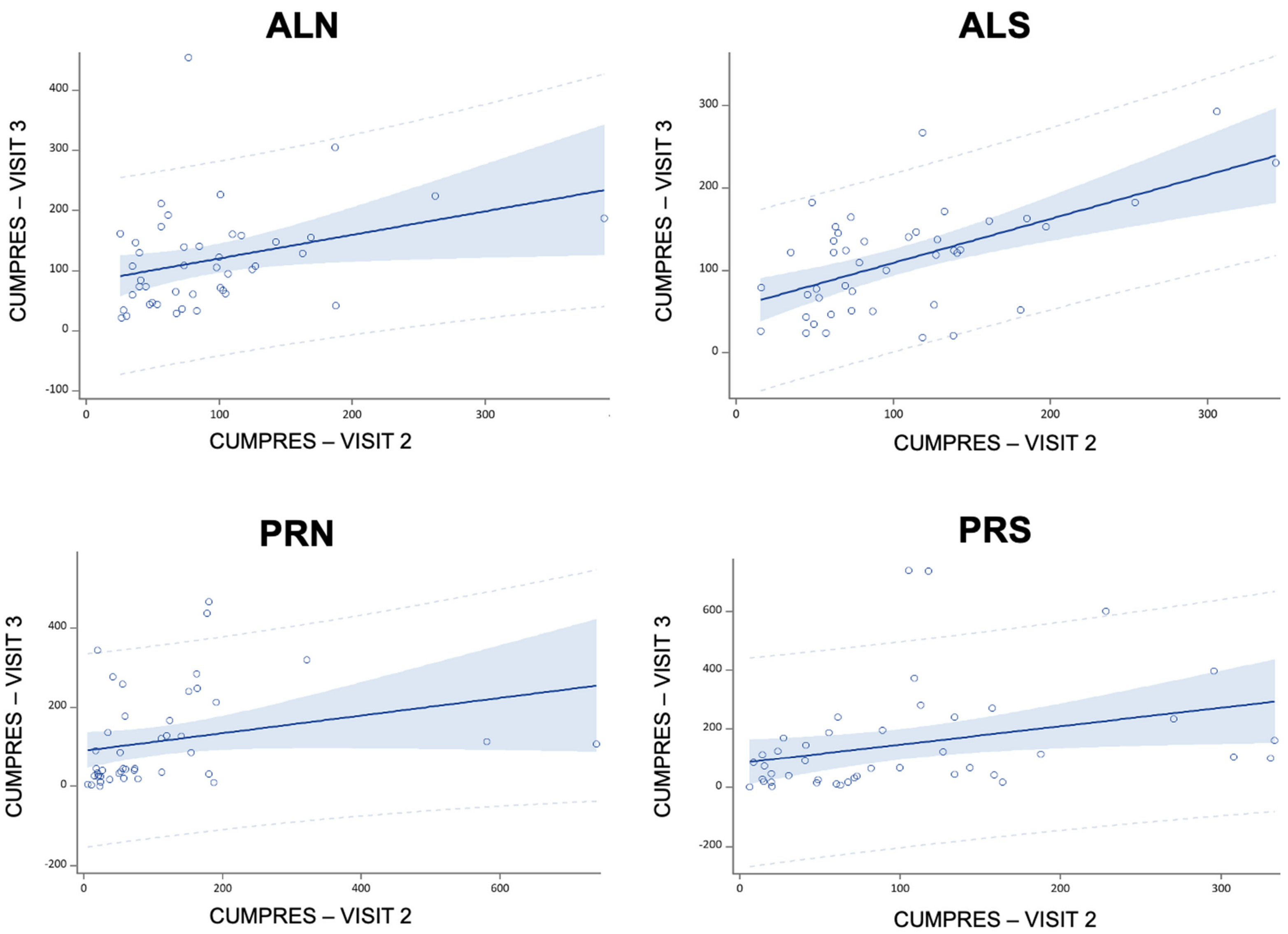

Supplementary Figure S2. Linear regressions of cumulative pressure (CUMPRES, psi) at visit 3 from visit 2 across conditions (schedule x beverage) for patients (*n* = 44). Statistics for each condition are presented in the text (Table 5). Each circle represents a single participant. The solid line represents the regression line and the dotted lines represent the 95% prediction interval. ALN, ad libitum schedule with non-sweet beverage. ALS, ad libitum schedule with sweet beverage. PRN, progressive ratio schedule with non-sweet beverage. PRS, progressive ratio schedule with sweet beverage.

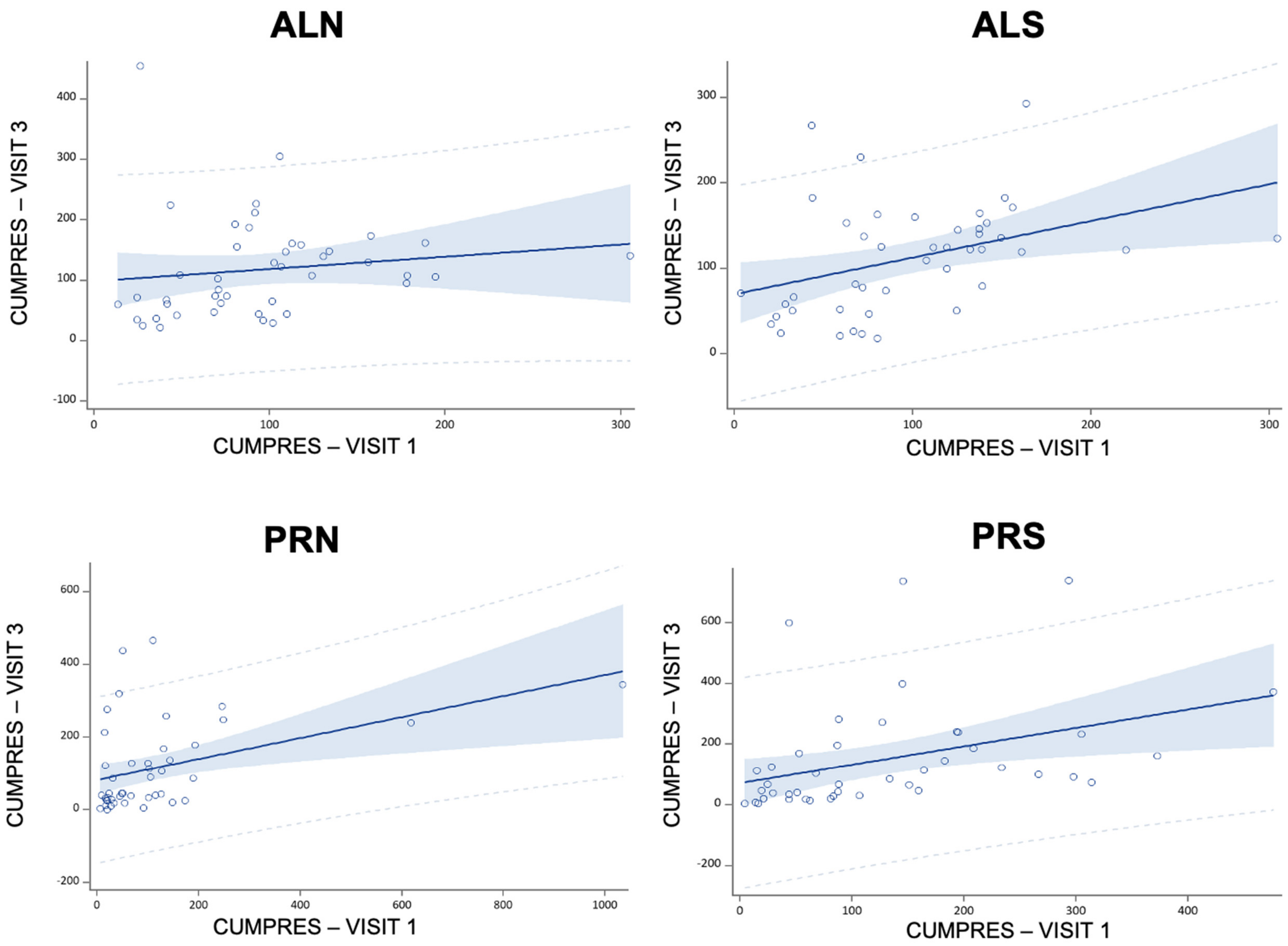

Supplementary Figure S3. Linear regressions of cumulative pressure (CUMPRES, psi) at visit 3 from visit 1 across conditions (schedule x beverage) for patients (*n* = 44). Statistics for each condition are presented in the text (Table 5). Each circle represents a single participant. The solid line represents the regression line and the dotted lines represent the 95% prediction interval. ALN, ad libitum schedule with non-sweet beverage. ALS, ad libitum schedule with sweet beverage. PRN, progressive ratio schedule with non-sweet beverage. PRS, progressive ratio schedule with sweet beverage.

SUPPLEMENTAL MATERIALS

Ad libitum (AL) Beverage Script

PLAY AUDIO

"When you are instructed to start, begin sipping on the straw as if you were drinking a beverage from a cup, and spit out into the container after each sip. The beverage will be delivered automatically to your mouth and you will need to sip in order to activate the delivery. Sip as much of the beverage as you want.

While you are sipping, you will be interrupted by a tone. When you hear the tone stop sipping.

Please wait in the room until the assistant returns to tell you the experiment is done.

Please restate the instructions about starting and stopping for the research assistant. If you have any questions, please ask now."

PAUSE AUDIO. ANSWER ANY QUESTIONS ABOUT INSTRUCTIONS. BEGIN AUDIO AGAIN

"The research assistant is going to leave the room now."

PRESS THE RED BUTTON BEFORE LEAVING THE ROOM. LEAVE THE ROOM.

ENTER COMPUTER ROOM to run program and monitor the subject

Press START on Matlab program
Make sure to do this before the subject starts sipping
Please do not begin sipping until this tape recording instructs you to start. Also, please do not at any time touch or move the box. Please do not rest your arms or elbows on the table, and do not push on the straw.

Please begin sipping NOW.

TONE after 2 minutes. When you hear the tone, RETURN TO THE ROOM and PRESS THE RED BUTTON.

If subject does not begin immediately, STOP the audio, and when subject starts sipping restart.

STOP SIPPING NOW.

PLAY AUDIO: "Please rinse your mouth out at least 3 times. This concludes this phase of the experiment."

Progressive Ratio Script

PLAY AUDIO

"When you are instructed to start, begin sipping on the straw. Sip as much of the beverage as you want but remember to spit into the container after each sip.

You will NOT hear a tone to stop for this trial Once you have stopped, please remain in the room and wait until the researcher returns. While waiting, if you decide you want to sip some more, you may do so.

At the end of the experiment, the researcher will return. Please restate the basic instructions about starting and stopping for the research assistant.

If you have any questions, please ask now.

PAUSE AUDIO. ANSWER ANY QUESTIONS ABOUT INSTRUCTIONS. BEGIN THE AUDIO AGAIN.

The research assistant is going to leave the room now.

PRESS THE RED BUTTON BEFORE LEAVING THE ROOM. LEAVE THE ROOM

ENTER COMPUTER ROOM to run program and monitor the subject

Press START on Matlab program.
Make sure to do this before the subject starts sipping

Please do not begin sipping until this tape recording instructs you to start.

ADD ENOUGH TIME TO START THE SIPOMETER
Please begin sipping NOW.

When the intake indicator stops changing start the stopwatch. Reset the stopwatch each time the indicator stops. When the stop watch reaches 2 min, instruct the subject to fill out the questionnaire.

If the subject rings the bell, wait a minute and if there is no further sipping, Go back in the room and press the RED BUTTON

After they finish, have the subject face the opposite wall and wait. Draw the curtain, and make necessary adjustments on the sipometer, e.g. switching the beverage container, spit container.

PLAY AUDIO: "Please rinse your mouth at least 3 times. This concludes this phase of the experiment. "
